# Pharmacological treatment of chorea in Huntington’s disease–good clinical practice versus evidence-based guideline

**DOI:** 10.1002/mds.25500

**Published:** 2013-05-14

**Authors:** Ralf Reilmann

**Affiliations:** 1 Huntington Group, Department of Neurology, University Clinic Muenster, Westfaelische Wilhelms University of MuensterMuenster, Germany

**Keywords:** chorea, therapy, Huntington’s disease, evidence-based medicine

## Abstract

Recently, the American Academy of Neurology published an evidence-based guideline for the pharmacological treatment of chorea in Huntington’s disease. Although the progress in medical care because of the implementation of criteria of evidence-based medicine is undisputed, the guideline classifies the level of evidence for drugs to reduce chorea based on anchors in the Unified Huntington’s Disease Rating Scale-Total Motor Score chorea sum score, which were chosen arbitrarily and do not reflect validated or generally accepted levels of clinical relevance. Thus, the guideline faces several serious limitations and delivers clinical recommendations that do not represent current clinical practice; these are reviewed in detail, and arguments are presented why these recommendations should not be followed. To remedy the lack of evidence-based recommendations and provide guidance to a pragmatic symptomatic therapy of chorea in HD, a flow-chart pathway that follows currently established clinical standards based on expert opinion is presented. © 2013 *Movement* Disorder Society

## Introduction: The Problem

Recently, the American Academy of Neurology (AAN) published an evidence-based guideline for the pharmacological treatment of chorea in Huntington’s disease (HD).^1^ The progress in medical care due to the implementation of criteria of evidence-based medicine is undisputed. The AAN guideline classifies the level of evidence for drugs to reduce chorea based on a review of randomized clinical trials that report data on Unified Huntington’s Disease Rating Scale-Total Motor Score (UHDRS-TMS) chorea scores.^2^ This interesting analysis may be valuable for further scientific and clinical discussion among experts, but it faces several serious limitations, which should caution a publication entitled “clinical guideline” that is suggestive of a clinical applicability of all recommendations presented.

A fundamental problem of the guideline is the assignment of “levels of importance” for different amplitudes of change in the UHDRS-TMS chorea sum score. These levels are used as anchors in the analysis, which classifies responses to therapy on which clinical recommendations in this guideline are based. A “2 to < 3-point” decrease in the UHDRS-TMS chorea sum score is classified as “moderately important,” whereas a “>3-point” decrease is considered “very important.” These cutoffs are arbitrarily chosen and do not reflect validated or generally accepted levels of clinical relevance for a change in the UHDRS-TMS chorea sum score. Seven subitems of different body regions contribute to the UHDRS-TMS chorea sum score. Thus, a mean improvement of just 1 point in less than 50% of the areas assessed could result in a “very important” change. Considering that chorea may not be the most relevant symptom of HD and that patients often do not seem to suffer from it,^3^ treatment recommendations based on these anchors are disputable. By the way, the UHDRS-TMS is a 124-point scale,^2^ not a 106-point scale, as claimed in the guideline, and it does not per se measure “parkinsonism.”

**Figure 1:**
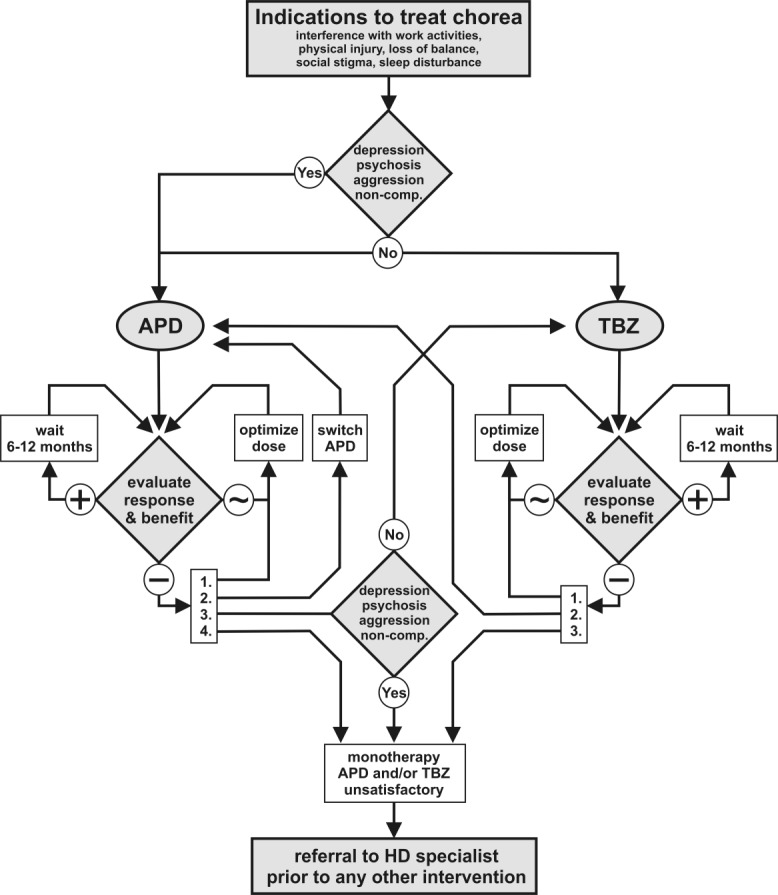
This flow-chart illustrates an easy to follow decision tree of different recommended treatments for chorea in Huntington’s disease (HD). Physicians may choose between antipsychotic drugs (APDs) (off-label) and tetrabenazine (TBZ). If depression, psychosis, aggressive behavior, or noncompliance are present, then TBZ should not be used. Different APDs may be explored before switching to TBZ. If monotherapy with either APDs or TBZ is unsatisfactory, then combination therapy should be considered, but it is recommended to refer patients to specialty centers to pursue this option (modified from Burgunder et al., 2011^4^).

Several specific recommendations for the use of drugs in treating chorea were given, which need a critical comment—these are presented in the “Caveat” sections below. In addition, a pragmatic approach to treating chorea in patients with HD, based on expert opinion, is presented in the form of a stepwise decision tree outlined in a flow chart (Fig. 1).

## Caveat 1: Riluzole

The recommendation to use riluzole to treat chorea, which is referred to even in the abstract of the guideline, is not clinically established.^4^ Indeed, 1 small phase II trial demonstrated a reduced UHDRS-TMS chorea sum score in patients who received riluzole 200 mg/day; however, this effect was not observed in the 100 mg/day arm of the study, and it did not survive a post-hoc analysis that excluded all patients who received concomitant neuroleptic medication in the 200 mg/day group.^5^ In addition, there was a relevant safety issue because of significant elevations of hepatic liver enzymes in the riluzole groups. Notably, the authors themselves concluded that, although riluzole appeared to have a mild antichoreic effect at the 200 mg/day dose, obstacles to its use included a lack of associated functional benefits and an ongoing need to monitor hepatic alanine aminotransferase levels. Thus, because of those limitations, the authors did not recommend the routine use of riluzole as antichoreic therapy. Incidentally, this was a small study that included about 20 patients in each arm, and, because of the variability of categorical clinical scales like the UHDRS-TMS, the results should be interpreted with caution. Thus, the study design itself should preclude these results from a direct translation into a clinical recommendation, as properly acknowledged by the investigators. Since then, a large phase III clinical trial did not demonstrate any effect of 100 mg/day riluzole on chorea.^6^ It is appreciated that the study was primarily designed to assess disease modifying effects. However, because of the sample size, it seems appropriate that post-hoc analyses, such as looking at changes in the UHDRS-TMS chorea sum score, are used as additional clinical evidence in the context of this discussion. It is noteworthy that, of 537 patients, two-thirds of whom were randomized (2:1) to treatment with riluzole for 3 years, only 379 completed the study. The main reason for discontinuation was the introduction of antichoreic medication, an observation underlining that no clinically relevant antichoreic effect of riluzole was observed in the study. Thus, the data available do not support the routine use of riluzole in HD and, accordingly, it is not prescribed by experts.^4^

## Caveat 2: Tetrabenazine

The uncritical use of tetrabenazine in doses of “up to 100 mg/day,” as recommended in the guideline, also is not clinically established practice.^4^ Although the authors rightly caution about possible side effects, such as parkinsonism and depression, it should be clearly emphasized that 100 mg/day is not a routinely desirable target dose. Tetrabenazine should be started at low doses (eg, 1–2 × 12.5 mg) and should be increased slowly and with care. In some countries, doses above 50 mg/day are avoided; and, in others, 75 mg/day is the recommended maximal dose. CYP2D6 (cytochrome P450, family 2, subfamily D, polypeptide 6) slow metabolizers are at risk of decreased metabolism of tetrabenazine and drug interactions; therefore, CYP2D6 genotyping is recommended for daily doses above 50 mg/day, eg, in the United States.^7^ Notably, tetrabenazine was associated with much discussed changes in secondary outcomes in the study that resulted in its approval: although the clinical global impression scale changed in favor of tetrabenazine, deleterious changes were observed in the UHDRS functional assessment scale, the Hamilton depression scale, and the Stroop word reading test of the UHDRS cognitive battery in the tetrabenazine group compared with placebo.^8^

## Caveat 3: Amantadine

In addition, the use of amantadine (300–400 mg/day), which is recommended in the first sentence of the guideline’s recommendation section right after tetrabenazine and before riluzole, requires a comment. The use of amantadine to treat chorea is highly controversial among experts, as recently revealed by a questionnaire obtained from HD experts: clinicians who had used amantadine described its benefit as small and transient; a minority considered it useful as monotherapy or adjunctive therapy, whereas a smaller but not insignificant number of respondents considered its use inappropriate for the treatment of chorea, and several cited inexperience with its use.^4^ The evidence for using amantadine in HD is sparse: although 1 trial reported a beneficial effect in a double-blind, placebo-controlled, cross-over design with 24 HD patients who received 400 mg/day,^9^ another study did not find a reduction of chorea in a similar sample size and design at 300 mg/day, although a semiquantitative patient questionnaire demonstrated a positive effect^10^; a meta-analysis of both studies, however, revealed no significant reduction in chorea.^11^ A very small, open-label series with less than 10 patients also reported a positive effect of amantadine in oral and intravenous formulations.^12^–^13^ The efficacy of intravenous delivery also was suggested by a small randomized controlled study.^14^ Nevertheless, these results are still inconclusive: accordingly, few experts use amantadine and, if they do, then it usually is not used in the first-line or second-line setting.^4^ Clearly, this evidence should not result in a “first-line” recommendation for using amantadine to treat chorea, as indicated even in the abstract of the AAN guideline.

## From Evidence-Based to Expert Opinion: Is There a Need for Both?

In conclusion, the guideline in its current form may be misleading and does not provide reference to treatment recommendations by experts or to standards established in common clinical practice.^4^–^15^ Uncritical application of this guideline may result in an increased incidence of side effects and potential harm to patients.

In part, this may be due to the fact that, in the process applied during the development of this AAN guideline, results of secondary endpoints from randomized clinical trials primarily designed to answer other questions were included. Although this may be useful for scientific discussion, clinical conclusions from these results may be biased and should not be directly translated into clinical guidelines. Accordingly, a recent Cochrane review applying criteria of evidence-based medicine concluded that no statement can be made regarding the best medical practice for the control of motor and nonmotor symptoms in HD.^11^

In this context, it should be considered that treatment recommendations in diseases with lower prevalence often lack the necessary clinical trials to document effects of clinically well-established therapies, as required by current evidence-based medicine criteria. Thus, expert opinions may be preferable to obtain meaningful guidelines.^4^ Although the authors of AAN guidelines are certainly aware of these limitations, it may be worth discussing whether formally correct analyzes like that under discussion here should be published as “guidelines”—a title suggestive of direct translatability into clinical practice.

Yet how can meaningful, less disputed recommendations be established and provide a readily accessible reference for clinical practice? Recently, a pragmatic pathway for treating chorea in HD was developed based on a questionnaire that was presented to 52 HD experts mainly from North America and Europe, as mentioned above.^4^ The results guided the design of a clinical pathway based on this expert experience; a slightly modified version is provided as flow-chart in Figure 1 to guide clinicians through the currently established options and criteria for selecting available pharmacologic interventions. In contrast to the original pathway, this revision includes the option to change from an antipsychotic drug (off-label) to tetrabenazine, or vice versa. It also forces a decision in favor of antipsychotics in case depression, psychosis, aggression, or noncompliance are present. Recommended antipsychotic drugs and their starting and maximal doses are listed in Table 1.

**Table 1:** Recommended first-choice antipsycotic drugs for the treatment of chorea in Huntington’s disease and their recommended starting doses and maximal doses^a^

APD of first choice	Respondents reporting, %	Recommended starting dose, mg	Recommended maximal dose, mg/d
Risperidone	43	0.5-2	16
Olanzapine	39	2.5–10	20
Tiapride^b^	29	50–200	900
Haloperidol	24	0.5-2	10
Quetiapine	12	25–200	400
Aripiprazole	11	2–15	30

a Doses were modified as reported by experts in the survey by Burgunder et al., 2011^4^ (modified). Modern APDs are preferred, although classical neuroleptics are used in several cases, usually with more severe phenotype.

b Tiapride is not available in all countries.

APDs, antipsychotic drugs.

This pathway will lead to satisfactory alleviation for most patients with clinically disturbing chorea who require treatment. It is important to bear in mind that treatment of chorea should aim to reduce involuntary movements, not to abolish them. Side effects, such as sedation and a negative impact on cognitive functioning, should always be assessed for and carefully monitored. Patients who respond inadequately to the suggested interventions should be referred to HD specialists. Currently, effective treatment of chorea should follow expert advice and not evidence-based guidelines in order to avoid side effects and potential harm to patients.

## Financial Disclosures

Dr. Reilmann has provided consulting services, advisory board functions, clinical trial services, quantitative motor analyses, and/or lectures for Novartis, Teva, Siena Bitoech, Neurosearch Inc., Medivation/Pfizer, Lundbeck, Ipsen, Wyeth, ISIS Pharma, Link Medicine, Prana Biotechnology, the Cure Huntington’s Disease Initiative Foundation USA, MEDA, Temmler Pharma, and AOP Orphan Pharmaceuticals AG. He serves on the Advisory Board of the “Jacques and Gloria Gossweiler Foundation,” he has several responsibilities in the European Huntington’s Disease Network (as a member of the “Executive Committee” and the “Clinical Trials Task Force” and as Lead Facilitator of the Neuroprotective Therapy and Motor Phenotype Working Groups). and he serves as Global Coordinating Principle Investigator of clinical trials. Dr. Reilmann has received grant support from the High-Q-Foundation, the Cure Huntington’s Disease Initiative Foundation (CHDI), the Deutsche Forschungsgemeinschaft (DFG), and the European Huntington’s Disease Network (EHDN).
